# Supplementary material for: A novel, rapid, quantitative method for face discrimination
Source: PLoS One. 2024 Dec 23;19(12):e0315998. doi: 10.1371/journal.pone.0315998 (PMC11666046; doi:10.1371/journal.pone.0315998)
Supplement: Attachment — Submitted filename: FaceDiscrimination_ResponseToReviewers.docx [file pone.0315998.s001.docx]

**Reviewer #1:**

The paper proposes and demonstrates a new efficient adaptive measure of face discrimination ability, by combining the authors' adaptive algorithm "FInD" with the smoothly-searchable PCA-based Basel Face space. The work seems soundly conducted. I have only a few suggestions for clarification:

MAJOR COMMENTS

- My one recurring point of confusion was how the separate structure vs texture components of the Basel Face Model were handled. The BFM consists of 199 dimensions capturing the 199 principal components (PCs) of variation in face mesh geometry, and another separate 199 dimensions capturing the 199 PCs of variation in RGB pixel values of the face texture. To visualise a face from the BFM, one specifies 398 numbers (the values of all structure and texture PCs), chooses a head angle and lighting, then renders the image. The structure and texture subspaces are the result of separate PCAs run on different data and don't have any relation to one another. In some places in the ms it sounds like only 199 numbers were specified, perhaps because the average texture (PCs = {0,0,0,....}) was applied to all faces, but then other parts of the ms seem to suggest that both subspaces were randomly sampled. Some sections in which to clarify this:
- line 136 "The faces are specified by 199 different parameters"
- line 139 "199 random values (...) for the structure and texture parameters"
- line 152 "All texture parameters are 0, only structure parameters are altered" (this seems clear - but comes a bit late, and seems to conflict with some of the other quoted parts)
- line 180 "same 199 random deviates (...) for the structure and texture parameters"

*Thank you, we have clarified the description in the revised manuscript in the section “Stimuli”.*

*In summary, we create unique random values for the 199 structural and textural Basel Face Model principal components for the face pair in each cell. The face pair within a cell share the same set of random values for 198 structural and 199 textural components; but component values differ across cells. Within each face pair, only a single target structural component differs, and all textural components remain constant. Therefore, the faces within a cell are similar (by a near-threshold distance), but faces between cells are very different..*

MINOR COMMENTS

- Following on from the previous point, it would generally be helpful to discuss the BFM more precisely in terms of PCs, rather than abstract "parameters". E.g. line 136-137 "199 different parameters each of which controls a different aspect of the face." This doesn't help a reader who isn't familiar with the BFM think about what sorts of "aspects" are being varied. It's also a bit confusing to readers who are familiar with the BFM, because there are 398 parameters per face really, or 199 *structural* parameters.

*Thank you, we have added text to explain that the BFM is a PC model with separate structural and textural components, and we use the term ‘component’ to refer to the parameters throughout the revised manuscript.*

- It is interesting to see the data in Figure 3 showing face discrimination thresholds for changes in each PC separately. The fact that early PCs induce larger visual changes is clear from playing with the BFM, but I haven't seen this quantified before. I would be curious to know whether the decrease in sensitivity is matched to the decrease in *physical stimulus change* created by varying each PC. This could be quantified either in the 3D space (as Euclidean distance between the original and altered face meshes), or image space (as pixelwise difference between the two rendered images).

*We agree that this is an interesting comparison, and we understand that principal components are already ordered according to the variance in the data. One of the goals of our paper was to compare the perceptual sensitivity with image variance captured in the BFM components.*

- Which version of the BFM does the work use? The issue is confusing because of the existence of the original morphable Basel Face Model in 2009, the collection of face photographs known as the Basel Face Database released in 2018, and then the new-and-improved morphable Basel Face Model version from 2019. From the figures it looks like the 2009 BFM (e.g. the face images are clearly computer-generated, so are BFM not BFD, and they have sharply cropped foreheads rather than visible hairlines pointing to 2009 rather than 2019 BFM). However, the authors reference Walker et al (2018) in several key places (e.g. Stimuli, p7), which I believe is a paper presenting the Basel Face Database, which is not a morphable model. And then the URL provided in the code repository to download the BFM points to the 2019 version (Gerig et al 2019 - which isn't cited). Please clarify throughout. It would also be good to include a brief discussion note on the distinctions between the 2009 and 2019 BFM version - e.g. could using the 2019 version help improve the diversity of the faces?

*We utilize the 2019 version of the BFM, this has been clarified in the text and the Gerig paper has been cited.*

- Are other researchers intended to use this method? The main goal of the paper seems to be to promote this task as an efficient method for measuring face discrimination ability. Code for the task appears online, suggesting that any researcher able to use PsychToolbox is free to run their own version of this experiment. However, the adaptive FInD algorithm underlying it is protected by a patent belonging to one of the authors. It would be good to clarify the status/intention - are researchers encouraged to use the freely available code, and meanwhile the algorithm may be built into a more user-friendly but paid app for clinicians or patients to use?

*We have clarified (Line 110) that the patent for* FInD *protects commercialization of the method, but researchers are free and encouraged to use the method for non-profit purposes. If the method can successfully quantify face processing differences (e.g. the presence or remediation of prosopagnosia), the longer-term goal would be to develop and distribute a user-friendly test.*

- line 57 "The (BFM) face space is isometric" - what is the evidence for this? In fact it seems clearly untrue as the data and examples in this paper show; moving 1 SD along an early principal component creates a much more visible change than moving 1 SD along a late component. It also creates a larger physical change in the stimulus (as mentioned above).

*The space is isometric in that equal Euclidean distance between face pairs renders them the same amount of “different” (see https://doi.org/10.1073/pnas.2115047119). However, we agree with the reviewer that moving along early PCs creates larger visual variation than moving along late PCs.*

*We have amended this statement (Line 55), which was originally cited from Blanz V & Vetter T (1999) & Paysan P et. al. (2009).*

- line 168 The power analysis mentioned here specifies a very large expected effect size (1 SD) - what is this the difference between, and in what statistical analysis? Was this effect size estimate arrived at prior to the experiment, and if so how?

*As this was an exploratory study, we ran a small pilot study that demonstrated robust effects. We have added some clarification in the main text.*

- line 316 It's not clear to me why this task would be better suited, in principle, to track changes in face perception, compared to other tasks?

*There are several features of this method that afford benefits over other tasks. Primarily the faces for each trial are unique, so the test can be repeated without confounds of stimulus learning or familiarity. Furthermore, the test avoids memory effects and recognition of known individuals. Ultimately, we propose that this task and could be it is a simple, rapid, effective could complement current methods that assess face perception.*

TYPOS etc

- line 26 "Thresholds measured remotely WERE not..."

*Thank you, corrected.*

- line 67 "there of" >> "thereof"

*Thank you, corrected.*

- line 96 what does the "participantive impact" of prosopagnosia mean?

*This word has been clarified to “participant’s perceived”.*

- line 115 "it's" >> "its"

*Thank you, corrected.*

- line 117 "stereoacuity (...), AND motion"

*Thank you, corrected.*

- line 166 "Additionally" - not clear why these participant demographics are reported separately

*Following NIH guidelines, the questionnaire contained a separate question for “Latino or Hispanic Origin”, distinct from “Race”, because individuals of Hispanic origin may be of any race.*

**Reviewer #2:**

This study explores human face discrimination thresholds using a novel approach. It employs the Basel Face Model to generate synthetic, parametrized face stimuli that are adaptively adjusted to estimate the discrimination thresholds specific to each BFM parameter. The main empirical finding is an inhomogeneity in how different BFM parameters relate to human face discrimination. This result advances the state of the art beyond what was previously described by Jozwik et al., 2022.

Unfortunately, this work relies on an unpublished and not fully disclosed adaptive psychophysical procedure, “FInD”. In my opinion, this omission raises significant concerns that must be addressed before the manuscript can be considered for publication. Additionally, I believe that there are several other major issues that must be corrected before the paper can be published, as described below.

Major
1.The FInD procedure, which is central to the current work, is not described in sufficient detail. The reference for the procedure is a VSS meeting abstract rather than a peer-reviewed paper. This issue becomes evident only after following the DOI link, as the reference initially appears to be a Journal of Vision paper.

Without a formal description and validation of the FInD procedure, many questions remain unanswered. For example: a. What Bayesian model is used? b. What assumptions does it make? c. How were the priors chosen? d. How is the efficiency metric calculated? e. What is the justification for Eq. 1? f. How is the potential interaction between cells taken (or not) into account? (The observer can quickly infer that there’s only a single positive cell and use this regularity in their decision-making). g. How reliable is the method compared to alternatives?

*There are now 2 peer-reviewed publications that use the* FInD *method and both are now cited in the revised manuscript.*

*a. The method doesn’t involve any Bayesian models,*

*b. The method only assumes d’ increases with signal intensity and that a decision boundary increases with d’ (see d. below)*

*c. There are no priors,*

*d. Efficiency is not calculated, but we have added repeatability data in a Bland-Altman analysis*

*e. We have added details to description of Equation 1to clarify that it is a saturating function of d’ as a function of stimulus intensity, based on computational (Foley & Legge, 1991) and physiological (Albrecht and Geisler, 1991) studies; followed by a decision stage (Gu and Green, 1994; Klein, 2004)*

*f. There are no assumptions about interactions between cells. The number of signal cells each chart is random, so the participant cannot infer anything about the probability that a given cell is a signal or a null cell based on their responses to other cells.*

*g. Based on our other studies, the FInD method is around TODO times faster than standard 2AFC psychophysics, depending on trial duration and the use of spatial or temporal alternatives.*

Without these details, fully evaluating this work is difficult. I suggest the authors either significantly revise the paper to include a complete description and validation of the FInD procedure or cite a detailed (preferably peer-reviewed) report on this method.

*Two additional peer-reviewed papers detailing the FInD method have been cited.*

2. The stimulus manipulation in Experiment 2 is not described in sufficient detail. What does it mean for the parameters to be manipulated in a combined fashion? Were both parameters changed to the same extent and in the same direction (i.e., both increased or both decreased)?

*When investigating combined parameters, two PCs were changed an equal amount. Specifically, target PC1 and target PC2 were both altered by the determined threshold deviation. Thus probability summation predicts an increase in sensitivity (decrease in thresholds) by a factor of √2.*

*This has been clarified in the text under “Experiment 2”.*

3. While the paper makes claims about the reliability of the method (e.g., “the use of an adaptive algorithm allowed the assessment across a range of ability levels with high precision”), it does not establish these claims. Failing to find significant differences between replications does not adequately demonstrate reliability, as null hypothesis testing does not control the type II error rate. Furthermore, for a potential diagnostic tool, the reliability of interest pertains to individual-level estimates rather than average estimates pooled from an entire group of subjects. I believe that to establish claims about reliability, measures such as test-retest reliability must be formally reported and compared to alternative approaches.

*We have performed an additional test-retest experiment and included a description and results at the end of the Results section.*

4. Judging from the VSS abstract, the model appears to be Bayesian, allowing for the calculation of a credible interval for each parameter estimate. Such intervals are not indicated, nor is any other measure of individual-level estimation error. Claiming that the estimation is “precise” without quantifying estimation error is problematic.

*The raw data from all charts are fit with Equation 1 after each chart using Matlab’s fit() function and we estimate confidence intervals for each participant. To avoid clutter in the figures, we show box and whisker plots for each figure and have added the range of 95% confidence intervals in the figure legend.*

Minor:
- Lines 253 and 256 seem inconsistent: “Thresholds for combined parameters were significantly lower than thresholds for single parameters”, versus “the overall average thresholds were lower for single parameters compared to combined in 7 out of 8 participants”.

*Thank you for noticing this semantic error, the secondary sentence has been amended such that single and combined are flipped.*

- Figure 3 displays horizontal stripes of measurements of equal threshold (around y=16). Is this an estimation artifact?

*Due to the PC nature of the Basel Face Model’s structure, the later PCs do not invoke as much change as early PCs. We start to see this banding in the later PCs (past 100), likely because this is the approximate mean for thresholds at these higher components.*

- It would be useful to spell out mathematically the predictions of probability summation in the current context.

*We expect the ratio of means between single and combined thresholds to exceed 1.4 for the result to point towards probability summation supperadditivity. We have clarified this in the text under “Single vs combined PC thresholds”.*

- Presenting figures at the end of the manuscript, disconnected from their captions, makes reviewing very cumbersome.

*Apologies, we are following the formatting guidelines of PLOS.*
