# Supplementary material for: A novel, rapid, quantitative method for face discrimination
Source: PLoS One. 2024 Dec 23;19(12):e0315998. doi: 10.1371/journal.pone.0315998 (PMC11666046; doi:10.1371/journal.pone.0315998)
Supplement: Attachment — Submitted filename: FaceDiscrimination_ResponseToReviewers2.docx [file pone.0315998.s002.docx]

**Reviewer #2:**

1. I appreciate the additional details on the FInD procedure. Unfortunately, the text remains insufficient for fully reproducing the experiment. Since the other papers cited are also applications of FInD rather than methods papers fully specifying the procedure, the method description in the current paper must be complete. For example, there is no mathematical specification of how the data is fitted. Is it maximum likelihood? Least squares fitting? Based on the defaults of MATLAB's fit() function (a function mentioned in the rebuttal but not in the paper), it appears to be the latter, but these details should be stated in the paper. Also, the source code shows that observation weighting (represented as 1./errEst) was applied, but this detail is not mentioned in the paper. Complete specification of the algorithm is especially important since the provided code is in MATLAB, a platform many researchers have transitioned away from in favor of Python or R. To ensure long-term reproducibility and accessibility to a broader scientific audience, the testing methods should be fully specified in the paper.

*We utilize the default fit() function with the default settings, which is the non-linear least squares method. The Matlab function* fittype() *is given Equation 1 to create a specialized fit model that is then used in* fit(). *These additional details have been added to the methods section.*

2. Bland-Altman analysis focuses on systematic bias and is typically used to assess the agreement between two measurement tools on average. Its application here seems misguided since the primary error component of interest in test-retest analysis is variance, not bias. The current analysis does not measure variance; unbiased yet completely random measurements would also fail to show significant differences. A more appropriate measure of reliability would be the linear correlation between the first and repeated measurements, calculated across participants, separately for each component. For the correlation coefficient to be meaningful, it should be compared to equivalent estimates obtained using alternative methods (e.g., 2AFC).

*We argue that Bland-Altman does test for systematic variance and is represented in the plots as horizontal blue lines. Additionally, we argue that correlation coefficients for test-retest are unreliable and overestimated when working with small sample sizes (<15), and Bland-Altman should be used instead.* *Some reasons why the Bland-Altman method is often preferred over the correlation coefficient for test-retest repeatability assessment include:*

- *Measure of Agreement vs. Association:*
  - *The correlation coefficient measures the strength of a linear relationship between two variables, but it does not assess agreement. High correlation can occur even if there is a systematic difference between measurements.*
  - *The Bland-Altman plot, on the other hand, directly assesses agreement by plotting the differences between two measurements against their averages. This method highlights any systematic bias and the limits of agreement, providing a clearer picture of repeatability.*
- *Detection of Bias:*
  - *The correlation coefficient does not reveal any bias between measurements. Two sets of measurements can have a high correlation even if one consistently overestimates or underestimates the other.*
  - *The Bland-Altman plot can detect both fixed and proportional bias, making it easier to identify and correct systematic errors.*
- *Handling of Measurement Error:*
  - *The correlation coefficient can be misleading if the measurement error is large relative to the variability between subjects.*
  - *The Bland-Altman plot accounts for measurement error by showing the spread of differences, which helps in understanding the precision and repeatability of the measurements.*

*Overall, while the correlation coefficient is useful for assessing the strength of a relationship, we believe the Bland-Altman plot provides a more comprehensive assessment of agreement and repeatability, making it a better choice for this test-retest reliability study. For reference, please see* [*https://support.sas.com/resources/papers/proceedings18/1815-2018.pdf*](https://support.sas.com/resources/papers/proceedings18/1815-2018.pdf)

*Regardless, we have performed a correlation analysis for each of the 10 individual components as per the reviewer’s request. Specifically, we found the linear correlation between the first and repeated measurements, across participants, separated across components. We found that the components 7 through 10 had significant correlations.*

3. Unless the authors establish reliability and efficiency empirically (e.g., as outlined in the previous point), I suggest the last sentence of the abstract, “Our results highlight the effectiveness and efficiency of this paradigm to measure face discrimination ability in-lab or remotely,” should be removed, as it is not directly supported by the analyses presented in the current study.

*As outlined above, we believe that the Bland-Altman analysis included in the manuscript establishes reliability in our paradigm.*
