# Supplementary material for: A novel, rapid, quantitative method for face discrimination
Source: PLoS One. 2024 Dec 23;19(12):e0315998. doi: 10.1371/journal.pone.0315998 (PMC11666046; doi:10.1371/journal.pone.0315998)
Supplement: Attachment — Submitted filename: FaceDiscrimination_ResponseToReviewers3.docx [file pone.0315998.s003.docx]

Reviewer #2: I appreciate the authors' detailed rebuttal. I have reviewed the reference provided by the authors and consulted additional sources.

I agree that the vertical spread in the Bland-Altman plot, quantified by the standard deviation of the differences, reflects the extent of measurement error. To determine whether the standard deviation is sufficiently small, it must be compared with a meaningful quantity: the magnitude of clinically significant differences, the error of an alternative measurement tool, or inter-subject variability. Since none of these comparisons were conducted, the Bland-Altman plots in Figure 8 cannot be used to assess test-retest reliability. Importantly, even a measure with zero test-retest reliability can still produce a benign Bland-Altman plot, showing no significant mean or slope differences and no outliers beyond two standard deviations. Therefore, the test-retest reliability subsection (lines 311–318) is misleading, as it does not evaluate reliability at all; instead, the tests reported assess systematic bias (difference mean) or error variance heterogeneity (difference slope).

I agree with the authors that linear correlation is positively biased for small samples and quantifies only reliability rather than agreement. This renders linear correlation a necessary but insufficient measure for reliability. As noted in the rebuttal, the test-retest linear correlation is not significantly different from chance for six out of ten components. Without test-retest reliability, we cannot have test-retest agreement.

Unlike the linear correlation, Lin's Concordance Correlation Coefficient (CCC) captures both variance and bias. I applied Lin’s CCC to component 1, using data from the rebuttal’s scatter plot. This yielded a value of approximately 0.02, indicating extremely poor agreement. This result heightens the concern that reliable measurement of individual, participant-specific thresholds has not been established.

I believe that the manuscript provides some interesting observations on the thresholds on average, across observers. Currently, it does not support its claims about precision, efficiency, or reliability of the measurement method itself at the individual level. The proposed method may be superior to traditional alternatives; however, the small sample size and low inter-subject variability among the 11 neurotypical participants likely limit our ability to confirm this. The individual level is important, as medical/neuropsychological diagnosis is explicitly mentioned as the general motivation for the proposed method (lines 78–88).

In my opinion, there are two potential directions for proceeding:

1. Rigorously study the reliability of the proposed method and its agreement with alternative tools by collecting a substantially larger participant sample (potentially including clinically relevant variability), analyzing test-retest agreement and between-method agreement using Lin’s CCC (in addition to Bland-Altman plots), and comparing both bias and variance with those of alternative measures (e.g., 2AFC).

*In other published works (cited below) we show that the FInD method is comparable to traditional 2AFC tasks – but because no face discrimination test like this currently exists, we cannot directly compare this face discrimination task to a similar 2AFC task. We agree that to confirm agreement with similar tests requires future work. Because of this we have rephrased the manuscript to focus on the novelty of the paradigm and have removed claims of efficacy and reliability.*

doi:10.1167/jov.22.14.4351

doi:10.1167/jov.21.9.2817

doi:10.1371/journal.pone.0305036

<https://iovs.arvojournals.org/article.aspx?articleid=2774587>

2. Rewrite the paper to focus on the trends shared across subjects. This would require removing all claims of efficacy and reliability, as well as the diagnostic framing and motivation. In that case, the title should be changed as well.

*We have removed claims of efficacy and reliability throughout the manuscript and have edited the title to focus on the novelty of this paradigm: “A novel quantitative assessment of face discrimination ability”.*
